# Data on the spatial distribution of 1,1-dimethylhydrazine and its transformation products in peat bog soil of rocket stage fall site in Russian North

**DOI:** 10.1016/j.dib.2020.105614

**Published:** 2020-04-23

**Authors:** Nikolay V. Ul'yanovskii, Dmitry E. Lakhmanov, Ilya I. Pikovskoi, Danil I. Falev, Mark S. Popov, Alexander Yu. Kozhevnikov, Dmitry S. Kosyakov

**Affiliations:** a Core Facility Center ‘Arktika’, Northern (Arctic) Federal University, Arkhangelsk, 163002, Russia; b Federal Center for Integrated Arctic Research of the Russian Academy of Sciences, Arkhangelsk, 163000, Russia

**Keywords:** 1,1-Dimethylhydrazine, Rocket fuel, Rocket stage fall place, Pollution, Transformation products, Spatial distribution, Peat bog soil

## Abstract

The data set covers the results of a study of 96 samples of peat bog soil from the fall place of the first stage of the Cyclone-3 launch vehicle contained unburned toxic rocket fuel 1,1-dimethylhydrazine (UDMH) in the European North of Russia. Soil samples were taken during a helicopter expedition to the “Koida” fall region of Plesetsk Cosmodrome operation zone in October 2015 at different distances from the center of the fall site and from different soil horizons. Samples were analyzed by liquid chromatography with amperometric detection and gas chromatography - tandem mass spectrometry. The contents of UDMH and the ten most important products of its transformations (methylhydrazine, hydrazine, 1,1,4,4-tetramethyltetrazene, formaldehyde, acetaldehyde and furaldehyde *N,N*-dimethylhydrazones, 1-formyl-2,2-dimethylhydrazine, *N,N*-dimethylformamide, *N*-nitrosodimethylamine, and 1-methyl-*1H*-1,2,4-triazole) were determined. The obtained data reflect the spatial distribution, migration and transformation of UDMH in the fall places of rocket stages under conditions of subarctic which is discussed in related research article “Migration and transformation of 1,1-dimethylhydrazine in peat bog soil of rocket stage fall site in Russian North” [1]. They can be further used for understanding the UDMH transformation pathways in soils rich in organic matter and assessment of environmental impact of space rocket activities in high latitudes.

Specifications Table

**Table:** 

Subject	Pollution
Specific subject area	Pollution of soil with highly toxic rocket fuel 1,1-dimethylhydrazine and its transformation products
Type of data	Table
How data were acquired	The quantification of hydrazines (UDMH, methylhydrazine, hydrazine) was carried out by hydrophilic interaction liquid chromatography with amperometric detection after preliminary steam distillation of analytes from soil samples. The instrument used is LC-20 Prominence HPLC system consisted of DGU-3 vacuum degasser, two LC-20ADsp pumps, SIL-20A autosampler (Shimadzu, Kyoto, Japan) and DECADE II electrochemical detector (Antec Leiden, Zoeterwoude, Netherlands). Labsolution software (Shimadzu, Kyoto, Japan) was used for data collection and processing. The quantification of other UDMH transformation products was carried out by gas chromatography – tandem mass spectrometry with preliminary pressurized liquid extraction of soil samples with acetonitrile. An Agilent 7000B GC-MS/MS system (Agilent, Santa Clara, USA) with triple quadrupole mass analyzer and autosampler was used. Mass Hunter software (Agilent, Santa Clara, USA) was used for data collection and processing.
Data format	Raw and analyzed data
Parameters for data collection	Soil samples were collected at the carrier rocket first stage fall site, immediately transported to laboratory in Arkhangelsk and frozen in airtight containers.
Description of data collection	Homogenized soil samples were subjected to steam distillation in strong alkaline medium into solution of sulfuric acid in acetonitrile (HPLC analysis of hydrazines) and to pressurized liquid extraction with acetonitrile after addition of excess barium hydroxide (for GC-MS/MS analysis of eight UDMH transformation products).
Data source location	Institution: Core Facility Center “Arktika”, Lomonosov Northern (Arctic) Federal University City: Arkhangelsk Country: Russia Latitude and longitude (and GPS coordinates) for collected samples: 66°10′14′' 43°01′06′'
Data accessibility	Repository name: Mendeley Data Data identification number: 10.17632/xxrfygp99g.1 Direct URL to data: http://dx.doi.org/10.17632/xxrfygp99g.1
Related research article	Nikolay V. Ul'yanovskii, Dmitry E. Lakhmanov, Ilya I. Pikovskoi, Danil I. Falev, Mark S. Popov, Alexander Yu. Kozhevnikov, Dmitry S. Kosyakov, Migration and transformation of 1,1-dimethylhydrazine in peat bog soil of rocket stage fall place in Russian North, Science of the Total Environment

## Value of the Data

• The data are useful to understand the processes of fate, migration and transformation of highly toxic 1,1-dimethylhydrazine rocket fuel in subarctic peat bog soils
• The data provide insights, which can be used by researchers, ecologists and physicians to assess the risks associated with the space rocket activities in high latitudes
• The data provide detailed information on concentration levels of 1,1-dimethylhydrazine and its 10 transformation products in peat bog soil, which is mandatory for the development of analytical methods and environmental monitoring system for the fall areas of launch vehicle spent parts.

## 1 Data Description

**Fig. 1:**
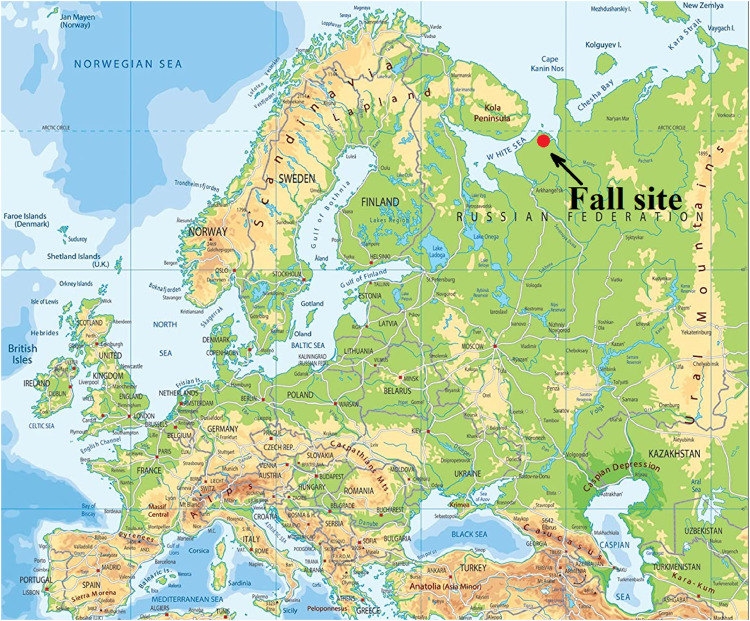
Location of the studied launch vehicle fall site on the map of Europe.

**Fig. 2:**
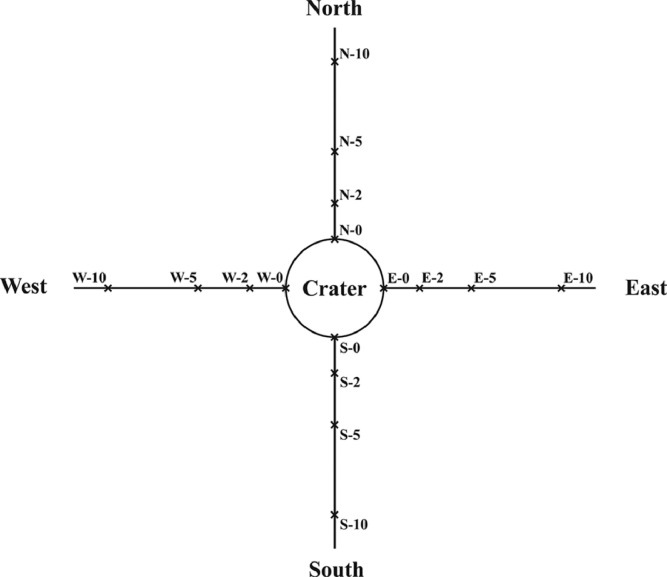
Sampling scheme and sampling points.

The data set covers the results of a study of 96 samples of peat bog soil from the fall site of the first stage of the Cyclone-3 launch vehicle (launched from Plesetsk Cosmodrome in 2004) contained unburned toxic rocket fuel 1,1-dimethylhydrazine (UDMH) in the European North of Russia (Fig. 1). The fall site is located in a peat bog and contains a round central crater (diameter of 6 m) filled with swamp water. Location of sampling points is presented in Fig. 2. Their designations are given in the following format: Cardinal direction (N, S, W, E) – distance from the central crater (m).

We present two data files in this article.

Data file 1 (Excel spreadsheet) presents the concentrations of hydrazines (UDMH; methylhydrazine, MT; and hydrazine, H) in soil samples determined by hydrophilic interaction liquid chromatography with amperometric detection after steam distillation of analytes from alkaline medium.

Data file 2 (Excel spreadsheet) presents the concentrations of eight UDMH transformation products (1,1,4,4-tetramethyltetrazene, TMT; formaldehyde *N,N*-dimethylhydrazone, DMHF; acetaldehyde *N,N*-dimethylhydrazone, DMHA; furaldehyde *N,N*-dimethylhydrazone, DMHFur; 1-formyl-2,2-dimethylhydrazine, FADMH; *N,N*-dimethylformamide, DMF; *N*-nitrosodimethylamine, NDMA; and 1-methyl-1H-1,2,4-triazole, MT) determined by gas chromatography – tandem mass spectrometry (GC-MS/MS) after pressurized liquid extraction of soil samples. The main parameters of GC-MS/MS analysis are presented in Table 1.

**Table 1:** Parameters of GC-MS/MS analysis of the UDMH transformation products

Analyte	Elemental composition	Retention time, min	Ion transition, *m/z*	Collision energy, eV	Ion transitions intensity ratio
DMHF	C_3_H_8_N_2_	2.3	72→71*	10	1.47
			72→57⁎⁎	10	
DMHA	C_4_H_10_N_2_	2.4	86→71*	10	1.04
			86→44⁎⁎	10	
TMT	C_4_H_12_N_4_	3.2	116→44*	7	2.47
			116→72⁎⁎	3	
NDMA	C_2_H_6_N_2_O	5.1	74→44*	5	2.57
			74→42⁎⁎	20	
DMF	C_3_H_7_NO	5.3	73→44*	5	4.15
			73→58⁎⁎	5	
MT	C_3_H_5_N_3_	8.7	83→56*	5	3.55
			83→28⁎⁎	20	
FADMH	C_3_H_8_N_2_O	9.6	59→44*	10	1.58
			59→29⁎⁎	20	
DMHFur	C_7_H_10_N_2_O	11.3	138→95*	10	1.34
			138→81⁎⁎	10	

⁎ ion transition for quantification

⁎⁎ ion transition for confirmation

The concentration values in both data files were calculated for initial wet soil samples and oven-dried ones using the data on moisture content also presented in the Excel spreadsheets. All concentrations are presented with standard deviation value calculated on the basis of three replicate analyses of each soil sample.

## 2 Experimental Design, Materials, and Methods

Soil sampling was carried out during a one-day helicopter expedition in October 2015. The samples were taken from the border of the crater (0 m) and also at the distances of 2, 5 and 10 m from it in the four cardinal directions (Fig. 2) using the Mole manual stainless steel soil sampling probe (Bürkle GmbH, Bad Bellingen, Germany) with combination drill bit and a handle with 175 cm rod. At each of 16 sampling points, the soil samples (∼ 1 kg each) were taken from six depths – 0–20, 20–40, 40–60, 60–80, 80–100, and 130–150 cm. Immediately after sampling, they were packaged in airtight polypropylene containers and, upon delivery to the laboratory on the same day, were deep frozen at –25°C. On the day of analysis, frozen soil samples were thawed at room temperature and thoroughly averaged by mixing with spatula.

For the extraction of hydrazines from soil samples, an approach based on the steam distillation of analytes from a strongly alkaline medium was used [2]. A 5-g weighed portion of soil was placed in a 250-mL round bottom flask and poured with 40-mL of a 50% aqueous solution of sodium hydroxide. The mixture was heated to boiling and distilled to dryness into receiving flask containing 10 mL of a 0.01 M solution of H_2_SO_4_ in acetonitrile. The obtained distillates were brought to a volume of 100 mL by adding acetonitrile, filtered through a membrane nylon filter (0.22 μm pore size) and injected into the HPLC system. To extract the other eight TPs, pressurized liquid extraction (PLE) with acetonitrile [3] in the ASE-350 automatic system (Thermo, Waltham, USA) with a working pressure of 100 bar was used. A soil sample (5 g) was thoroughly mixed with barium hydroxide in a ratio of 2.5 g of alkali per 1 g of soil (recalculated to oven-dried substance) and placed in a 10 mL stainless steel extraction cell. A mixture of acetonitrile with water (9:1) was used as an extractant. Extraction parameters were as follows: temperature 100°C, number of cycles - 2, duration of one cycle 10 min, the final washing of the sample with a fresh portion of the solvent (60% of the cell volume). The total extraction time of the sample was 30 min, the volume of the obtained extract was 25–30 mL. In order to prevent contamination of the ASE communications with barium hydroxide the system was additionally washed with a 3% aqueous solution of acetic acid after the extraction of each sample.

The quantification of UDMH, MH and H in the obtained soil distillates was carried out by HPLC with amperometric detection in accordance with [4]. An LC-20 Prominence HPLC system (Shimadzu, Kyoto, Japan), consisting of an LC-20 ADsp pump, a SIL-20A autosampler, a CTO-20A column thermostat, a DGU-20A5R vacuum degasser, a CBM-20 controller, and a DECADE II electrochemical detector (Antec Leiden, Zoeterwoude, Netherlands) was used. The chromatographic separation of analytes was achieved at 40°C in hydrophilic interactions liquid chromatography (HILIC) mode on a Nucleodur HILIC column (Macherey-Nagel, Duren, Germany), 150 × 3.0 mm, particle size 3.0 μm, with a zwitterionic sulfobetaine stationary phase. A mixture of 20 mM phosphate buffer solution (pH 2.5) and acetonitrile (22:78 v/v) was used as a mobile phase in isocratic elution mode with the flow rate of 0.5 mL min^–1^. The volume of the injected sample was 10 μL. Detection was carried out on a glassy carbon working electrode at a potential +1.1V; a glass pH sensitive electrode acted as a reference electrode. Instrument control, data collection and processing were performed using LabSolution software (Shimadzu, Kyoto, Japan).

Direct analyses of PLE soil extracts by GC-MS/MS [5] were performed on an Agilent 7000B gas chromatography–tandem mass-spectrometry system (Agilent, Santa Clara, USA), which consisted of an Agilent 7890A gas chromatograph and a triple quadrupole mass spectrometric detector. Separation was achieved on an HP INNOWax capillary column (Agilent, Santa Clara, USA), 30 m × 0.25 mm, film thickness 0.25 μm. The operation parameters of the GC-MS/MS system were as follows: helium carrier gas (99,9999%, NIIKM, Moscow, Russia), constant pressure (103 kPa) mode, electron ionization (70 eV), injector temperature 170°C interface temperature 230°C, ion source temperature 230°C, voltage on the detector 0.8 kV (automatic adjustment). The gas in the collision cell of the mass spectrometer was nitrogen. The injected volume was 2 μL with split 5:1. Oven temperature was programmed from 100°C to 190°C at the 10°C min^–1^ ramp. To clean the chromatography column after the analysis, it was kept at 230°C for 3 min. The total analysis time was 12 min. Mass spectrometric detection was performed in the multiple reaction monitoring (MRM) mode with the parameters listed in Table 1.

The control of the GC-MS/MS system, the acquisition and processing of the data were accomplished using the MassHunter software (Agilent, Santa Clara, USA).

The limit of detection (LOD) values (µg kg^–1^) for analytes in soil (recalculated to oven-dried sample) were as follows: UDMH – 20, MH – 26, H – 14, DMHF – 2.4, DMHA – 3.8, TMT – 4.0, NDMA – 1.8, DMF – 5.8, MT – 2.0, FADMH – 14, DMHFur – 15.

## Appendix Supplementary materials

Image, application 1
